# Effect of yoga and music on depression among antenatal Indian women

**DOI:** 10.6026/973206300211458

**Published:** 2025-06-30

**Authors:** Rohit Divya Gunvanthbhai, Siva Subramanian N., Mahalakshmi B.

**Affiliations:** 1 Depaertment of Obstetrics & Gynaecological Nursing, Sankalchand Patel university, Visnagar, Gujarat - 384315; India; 2 Department of Psychiatric Nursing, Nootan College of Nursing, Sankalchand Patel University, Visnagar, Gujarat - 384315; India; 3 Department of Paediatric Nursing, Nootan College of Nursing, Sankalchand Patel University, Visnagar, Gujarat - 384315; India

**Keywords:** Antenatal depression, yoga therapy, music therapy maternal mental health

## Abstract

Antenatal depression is a growing concern affecting maternal and fetal health, often left untreated due to stigma and limited resources. Therefore, it is of interest to evaluate the effectiveness of yoga and music therapy in reducing depression among pregnant women in urban Gujarat. Hence, a total of 180 antenatal women were assigned to either a yoga group (2-week intervention) or a music group (1-week intervention). Depression levels were assessed pre- and post-intervention using the Edinburgh Postnatal Depression Scale. The yoga group showed a significant reduction in depression scores (p < 0.001), while the music group showed a slight but non-significant improvement (p = 0.369). Results show that yoga is more effective than music in alleviating depressive symptoms during pregnancy.

## Background:

Depression during pregnancy is a widespread mental health issue, affecting up to 20.7% of women worldwide and contributing to adverse outcomes such as preterm birth, low birth weight, and developmental delays in children [1]. Despite clinical recommendations for screening, many women receive no treatment due to social stigma, cost, or limited access to psychological care [2]. This has led to increased interest in complementary therapies such as yoga and music, which are considered safe, low-cost, and culturally acceptable options for mood regulation [3]. Yoga, a blend of postures, breathing techniques, and mindfulness, has shown notable benefits for emotional regulation. A systematic review by Gong *et al.* reported a statistically significant reduction in depression scores among pregnant women practicing integrated yoga forms (including meditation and pranayama) [4]. Similarly, Lin *et al.* found that yoga was particularly effective for women already experiencing clinical depression, leading to improvements in both depression and anxiety scores [5]. Music therapy has also demonstrated promise in maternal mental health care. In a comparative meta-analysis, Zhu *et al.* found that music was among the most effective non-pharmacological interventions for antenatal depression, especially when culturally relevant forms were used [6, 7]. Therefore, it is of interest to assess the effect of yoga and music on depression among antenatal Indian women.

## Methodology:

## Research design and setting:

A quantitative, quasi-experimental design was employed to evaluate the effectiveness of complementary therapies-Yoga and Music-on depression among antenatal women. The study was conducted in selected urban areas of Gujarat, India.

## Population and sampling:

The study population included antenatal women between the ages of 19-35 years. A total of 180 participants were selected using a convenience sampling technique, with 90 women in each group (Yoga and Music). Women with mild to moderate depression and low to moderate stress, as measured by validated scales, were included. Women with high-risk pregnancies or under psychiatric treatment were excluded.

## Intervention and tool:

The Yoga Therapy Group participated in 30-minute daily sessions for two weeks, focusing on gentle prenatal asanas, breathing techniques, and loosening movements tailored for pregnancy; the Music Therapy Group listened to calming instrumental music for 30 minutes daily over one week in a relaxed environment. Data collection tools included a Demographic Questionnaire to gather participant background information and the Edinburgh Postnatal Depression Scale (EPDS) to assess depression levels pre- and post-intervention.

## Data collection procedure:

After obtaining informed consent, a pre-test was conducted for both groups using the EPDS. Following the intervention period (two weeks for yoga, one week for music), a post-test was administered using the same scale to evaluate the change in depression levels.

## Ethical considerations:

Ethical approval was obtained from the institutional ethics committee. Informed consent was collected from all participants, and confidentiality was maintained throughout the study.

## Statistical analysis:

Descriptive statistics were used to summarize demographic data. Paired sample t-tests assessed within-group differences (pre- vs. post-test), and independent t-tests evaluated between-group differences. Pearson's correlation coefficient was used to examine the relationship between the therapies and depression scores. A p-value < 0.05 was considered statistically significant.

## Results:

Table 1 (see PDF) shows that most participants were aged 22-25, from middle-class families, and unemployed. The Yoga group had more postgraduates and nuclear families, while the Music group had more joint families. Primigravida women were the majority in both groups, with very few reporting previous abortions. Figure 1 (see PDF) shows that in the Yoga group, mild depression increased from 44.4% to 71.1% and moderate depression decreased from 55.6% to 28.9%. In the Music group, mild depression increased from 67.8% to 82.2% and moderate depression decreased from 32.2% to 17.8%, indicating a greater impact in the Yoga group. Table 2 (see PDF) show that Depression scores significantly decreased in the Yoga group (p < 0.001), while the Music group showed a small, non-significant change (p = 0.369), indicating yoga was more effective in reducing depression. Table 3 (see PDF) shows there was a weak positive correlation between depression reduction and yoga therapy (r = 0.030), while a moderate positive correlation was observed with music therapy (r = 0.346, p < 0.001), suggesting both therapies influenced depression, but differently.

## Discussion:

The present study was conducted to assess and compare the effectiveness of yoga and music therapy in reducing antenatal depression among pregnant women residing in selected urban areas of Gujarat. Our study shows that yoga therapy significantly reduced depression in pregnant women, with post-test scores dropping from 15.33 to 11.55 (p < 0.001). This finding supports the effectiveness of yoga as a non-pharmacological intervention for managing antenatal depression. Similar to our findings, a meta-analysis demonstrated a significant reduction in depressive symptoms following yoga-based interventions [8]. Supporting this, another study found that yoga participants showed greater improvements in depression than a parenting education group [9]. In line with our results, prenatal yoga was also reported to be widely used and perceived as beneficial for reducing mood disturbances [10]. Consistent with this, an 8-week yoga intervention significantly reduced negative affect among pregnant women [11]. A recent meta-analysis confirmed that prenatal yoga effectively reduced cortisol levels, improving emotional outcomes during pregnancy [12]. Echoing our findings, another systematic review emphasized that prenatal yoga positively impacted mental health outcomes, including stress and depression [13]. Our study also shows that music therapy led to a reduction in depression scores among pregnant women, though the change was not statistically significant (p = 0.369). This suggests that while music therapy has some positive emotional effects, its impact on clinical depression may be more modest compared to yoga. Consistent with our findings, a randomized controlled trial reported significant decreases in EPDS scores after two weeks of music intervention in pregnant women [14]. Corroborating this, another study found that listening to music during pregnancy enhances psychological well-being and reduces postpartum depression symptoms [15]. Similar outcomes were observed in a study that linked regular music listening to fewer depressive symptoms in the early postpartum period [16]. Further, music therapy was associated with significant improvements in depression and sleep quality among third-trimester women [17]. In line with this, a literature review concluded that music therapy is a safe, effective, and cost-free method to reduce stress and depression during pregnancy [18]. Finally, a comparative study found that a combination of yoga and music produced the most positive emotional outcomes, while standalone music therapy still showed strong benefits [19].

Additionally, although our study found yoga to be more effective in reducing average depression scores, music therapy had a moderate, statistically significant correlation with depression reduction (r = 0.346, p < 0.001), while yoga showed a weak, non-significant correlation (r = 0.030). This suggests that music therapy, while less impactful in terms of average reduction, provided a more consistent individual response. Supporting this, one study found that 84% of depressed pregnant women expressed strong interest in using music and yoga, with music often perceived as more accessible [20]. Similarly, a post-disaster recovery study found that music and massage were significantly associated with improved mental health outcomes during pregnancy [21]. A systematic review confirmed that music and massage therapies had more consistent benefits than yoga in reducing antenatal depression [22]. This was echoed by an umbrella review on complementary therapies, which highlighted moderate-to-strong correlations between music-based interventions and improved peripartum mental health [23]. A broader clinical review also concluded that music therapy had stronger emotional outcome correlations than yoga or supplements [24]. Furthermore, while yoga therapy resulted in a statistically significant reduction in depression (p < 0.001) and music therapy showed a smaller, non-significant improvement (p = 0.369), broader research suggests that music may be equally or more effective than yoga in specific contexts. A large meta-analysis comparing multiple interventions concluded that music therapy had the strongest overall impact on antenatal depression among all non-pharmacological treatments reviewed [25, 26]. Another comparative study found that combining yoga with music produced the best outcomes, but even music alone outperformed yoga in certain emotional domains [19]. In contrast, yoga therapy requires sustained engagement to achieve meaningful results, as supported by other reviews [27]. In conclusion, the study highlights yoga therapy as a more effective complementary approach than music therapy in reducing antenatal depression. While music therapy showed a moderate positive correlation and emotional benefit, the consistent and statistically significant impact of yoga suggests its superior potential in clinical application. These findings reinforce the need to integrate structured yoga programs into routine antenatal care services, especially in low-resource urban settings, to improve maternal mental health outcomes. Future research could explore the combined effects of yoga and music therapy, long-term benefits, and their adaptability across diverse populations.

## Conclusion:

Antenatal depression is a growing concern affecting maternal and fetal health, often left untreated due to stigma and limited resources. The yoga group showed a significant reduction in depression scores (p < 0.001), while the music group showed a slight but non-significant improvement. Results show that yoga is more effective than music in alleviating depressive symptoms during pregnancy.
